# Vancomycin dosing in critically ill patients receiving renal replacement therapy: a critical appraisal of the toxicity threshold and external validation

**DOI:** 10.1186/s13054-026-06322-0

**Published:** 2026-09-22

**Authors:** Jiaojiao Zhou, Haibo Lei, Ronghui Li, Xiang Liu, Guanghui Chen

**Affiliations:** Department of Clinical Pharmacy, The Central Hospital of Xiangtan (The Affiliated Hospital of Hunan University), Heping Road, Xiangtan, 411100 China

Dear Editor,

We read with great interest the article by Ulldemolins et al. [1] describing vancomycin dosing nomograms for critically ill patients receiving renal replacement therapy (RRT). The authors are to be commended for a large, multicentre pharmacokinetic study with rich sampling and for addressing a clinically important problem. However, we have several concerns regarding the toxicity threshold used for dosing simulations and the completeness of the external validation reporting, which may affect the clinical safety and applicability of the proposed nomograms.

First, and most critically, the toxicity threshold used in the dosing simulations is inconsistent with the cited guideline. The authors define the toxicity threshold as an area under the concentration–time curve over 24 h (AUC_0−24 h_) ≥ 700 mg·h/L, stating that this is “in line with current clinical guidelines” and citing the 2020 ASHP/IDSA/PIDS/SIDP vancomycin therapeutic monitoring guideline [2]. However, that guideline explicitly recommends that “daily vancomycin AUC values (assuming a MIC of 1 mg/L) should be maintained between 400 and 600 mg·h/L to minimize the likelihood of nephrotoxicity and maximize efficacy” for methicillin-resistant *Staphylococcus aureus* infection [2]. By expanding the therapeutic window from 400-600 to 400–700, the authors’ Monte Carlo simulations would classify dosing regimens with AUC values between 600 and 700 mg·h/L as “non-toxic,” whereas the cited guideline recommends maintaining AUC below 600 mg·h/L to minimise nephrotoxicity. Given that the study population already has severe acute kidney injury requiring RRT, this is not a trivial issue. We urge the authors to re-run their simulations using an upper limit of 600 mg·h/L and to reassess whether the proposed nomograms remain optimal.

Second, the reporting of the external validation results may not fully reflect the model’s performance across different patient populations. In the abstract, the authors state that “predictive performance was high (median prediction error = − 13.4%)”, which derives from the rich sampling validation dataset (*n* = 19). However, the manuscript also presents a separate TDM validation dataset (*n* = 27, 104 pre-filter concentrations), in which the a priori population predictions yielded a median prediction error of 81.9% and a median absolute prediction error of 81.9%. This indicates a systematic overprediction of vancomycin concentrations in that dataset. According to the authors’ own predefined acceptability criteria (MPE ≤ ± 20%, MAPE ≤ 30%, F20 ≥ 35%, F30 ≥ 50%), this a priori predictive performance falls outside the acceptable range. Notably, 46.7% of patients in the TDM dataset had urine output ≥ 500 mL/24 h during the first 48 h, compared with 21.5% in the development dataset, suggesting that the model’s bias may be related to residual renal function [3, 4]. If the nomogram is applied without therapeutic drug monitoring in patients with preserved urine output, the model’s tendency to overpredict concentrations could lead to underdosing. We therefore believe it would be helpful for the abstract and conclusions to clearly acknowledge the a priori prediction limitations observed in this external dataset, and for the nomogram to include a note of caution regarding its use in patients with significant residual urine output.

Third, beyond the reporting issues discussed above, the nomogram itself does not incorporate residual urine output as a stratification variable, despite its recognised influence on vancomycin clearance in this population [3, 4]. The authors acknowledge this limitation in the discussion, but the abstract and the nomogram do not reflect it. In clinical practice, critically ill patients receiving RRT may have recovering renal function or non-oliguric acute kidney injury, and applying a nomogram derived predominantly from oligoanuric patients to such individuals could result in subtherapeutic exposure. We would therefore suggest that the authors either add urine output as a stratification variable in the nomogram or explicitly state that the nomogram is intended only for oligoanuric patients and that therapeutic drug monitoring should be considered mandatory when urine output exceeds 500 mL/24 h.

In summary, we believe the study provides valuable pharmacokinetic data, but the dosing nomograms should be interpreted with caution. The toxicity threshold should be corrected to 600 mg·h/L in accordance with the cited guideline, and the external validation results should be fully and transparently reported. We would also welcome a sensitivity analysis using the threshold of 600 mg·h/L to further clarify whether the proposed regimens maintain an acceptable balance between efficacy and toxicity.

## Data Availability

No datasets were generated or analysed during the current study.
